# Outbreak of haemolytic uraemic syndrome in Norway caused by *stx*_2_-positive *Escherichia coli *O103:H25 traced to cured mutton sausages

**DOI:** 10.1186/1471-2334-8-41

**Published:** 2008-04-03

**Authors:** Barbara Schimmer, Karin Nygard, Hanne-Merete Eriksen, Jørgen Lassen, Bjørn-Arne Lindstedt, Lin T Brandal, Georg Kapperud, Preben Aavitsland

**Affiliations:** 1 Department of Infectious Disease Epidemiology, Norwegian Institute of Public Health, Oslo, Norway; 2 European Programme of Intervention Epidemiology Training (EPIET), Swedish Institute for Infectious Disease Control, Solna, Sweden; 3 Department of Food-borne Infections, Norwegian Institute of Public Health, Oslo, Norway; 4 Norwegian School of Veterinary Science, Oslo, Norway

## Abstract

**Background:**

On 20–21 February 2006, six cases of diarrhoea-associated haemolytic uraemic syndrome (HUS) were reported by paediatricians to the Norwegian Institute of Public Health. We initiated an investigation to identify the etiologic agent and determine the source of the outbreak in order to implement control measures.

**Methods:**

A case was defined as a child with diarrhoea-associated HUS or any person with an infection with the outbreak strain of *E. coli *O103 (defined by the multi-locus variable number tandem repeats analysis (MLVA) profile) both with illness onset after January 1st 2006 in Norway. After initial hypotheses-generating interviews, we performed a case-control study with the first fifteen cases and three controls for each case matched by age, sex and municipality. Suspected food items were sampled, and any *E. coli *O103 strains were typed by MLVA.

**Results:**

Between 20 February and 6 April 2006, 17 cases were identified, of which 10 children developed HUS, including one fatal case. After pilot interviews, a matched case-control study was performed indicating an association between a traditional cured sausage (odds ratio 19.4 (95% CI: 2.4–156)) and STEC infection. *E. coli *O103:H25 identical to the outbreak strain defined by MLVA profile was found in the product and traced back to contaminated mutton.

**Conclusion:**

We report an outbreak caused by a rare STEC variant (O103:H25, *stx*_2_-positive). More than half of the diagnosed patients developed HUS, indicating that the causative organism is particularly virulent. Small ruminants continue to be important reservoirs for human-pathogen STEC. Improved slaughtering hygiene and good manufacturing practices for cured sausage products are needed to minimise the possibility of STEC surviving through the entire sausage production process.

## Background

Shiga toxin producing *E. coli *(STEC) can cause bloody diarrhoea which in 2–15% of cases, particularly in children, develop into haemolytic uraemic syndrome (HUS) which can lead to renal failure and death [1]. More than 90% of diarrhoea-associated HUS cases are due to STEC infections. Routine diagnosis and surveillance of STEC-infections was originally developed for serotype O157:H7 of STEC. However, non-O157 *E. coli *infections are in certain geographic regions considered to be at least equally important, but may in general be underdiagnosed [2].

Sporadic STEC infections may be transmitted through food, contact with animals or farming environments or by person-to-person, the last two affecting mainly young children [3]. Outbreaks are mainly foodborne, and have been associated with a wide variety of products, including undercooked minced beef, unpasteurized milk or apple juice, yoghurt, cheese, lettuce, vegetables, cured sausages and drinking water [1].

In Norway (population 4.6 million), around 10 to 20 cases of sporadic STEC infection are notified annually. Most have only bloody diarrhoea [4], and approximately half have been acquired in Norway. The only documented foodborne STEC outbreak in Norway occurred in 1999 with four confirmed cases of *E. coli *O157:H7 infection, most likely caused by contaminated domestically produced lettuce [5].

On 20–21 February 2006, a cluster of four diarrhoea-associated HUS cases was reported to the Norwegian Institute of Public Health (NIPH) from an academic hospital in Oslo. Enquiries to other hospitals in Norway identified two additional HUS cases diagnosed since the beginning of 2006. Since hospital episode statistics indicate less than a handful HUS cases in children per year in Norway, we suspected an outbreak and launched an investigation in order to identify the source and stop the outbreak.

## Methods

### Epidemiological investigation

#### Case definition and case finding

For the outbreak investigation we defined an outbreak-related case as a child less than 16 years old, hospitalised in Norway with diarrhoea-associated HUS or a person of any age with an infection with the outbreak strain of *E. coli *O103 (defined by a specific multi-locus variable number tandem repeats analysis (MLVA) profile), both with onset after January 1, 2006. We alerted all medical microbiological laboratories and clinicians, in particular paediatric nephrologists, of the outbreak and requested rapid reporting of suspected cases.

#### Hypotheses-generating interviews

We interviewed all cases (or, if <16 years of age, their parents) in person or by phone with our standard 14-paged structured hypothesis-generating questionnaire covering clinical symptoms, demographics, food and drinks consumed, animal contacts and environmental exposures in the seven days preceding onset of diarrhoea. Most cases were contacted repeatedly to obtain a complete picture of their exposures. Local public health doctors or district food safety authorities interviewed school and kindergarten staff regarding foods eaten by cases in these locations and forwarded the data to the NIPH.

#### Case-control study

The case-control study started on February 22 and included all cases reported to the NIPH. The study was designed to measure association between disease and consumption of 26 food items that were suspected based on the hypothesis-generating interviews, including several types of meat, dairy products and vegetables, some of which were specified with brand names. Three controls per case were randomly selected from the computerised national population register matched by (closest) date of birth, gender and municipality of residence. For the cases, consumption of food items was asked for the period of one week before the onset of diarrhoea; the matched controls answered for the corresponding period. Controls that had travelled abroad during this period, or that did not speak Norwegian were excluded. Trained personnel interviewed cases and controls by telephone.

On February 24, with data from the first 6 cases and their 18 matched controls, the analysis indicated minced meat of brand A as the source. We reported this to the National Food Safety Authority (NFSA) and then continued the investigation. On March 14, when case number 11 was confirmed, hypothesis-generating interviews led us to suspect another product. We then extended the case-control questionnaire to include this product and interviewed all cases and controls also with the revised questionnaire. The second analysis of the case-control data included fifteen cases known by March 17. Results were reported on March 20 to the NFSA.

#### Statistical analysis

Interview data were entered in Microsoft Office Excel 2003. Answers were coded 'yes', 'no' or 'unknown'. Unknown was coded as missing in the analysis. The descriptive analysis and the conditional logistic regression models were performed in STATA 9.2 (Stata Corporation, College Station, TX, USA). Bivariate conditional regression analyses were initially run for all exposures using Wald chi-square test for significance. Risk factors with a p-value <0.2 were included in a multivariate conditional logistic regression model. Only factors to which more than half of the cases had been exposed, were considered in the model. We removed from the model one exposure at a time using the Wald p-value <0.1 as the elimination criterion. The results are reported as matched odds ratios (mORs) with 95% confidence intervals and two-tailed Wald p-values for the regression coefficients. Because of the small numbers of subjects in the first case-control study, it was not possible to obtain a conditional maximum-likelihood estimate of the odds ratio for all exposures. For these exposures, we calculated the median unbiased estimate (MUE) of the odds ratio and exact 95% confidence intervals using LogXact 4 (Cytel Software Corporation, Cambridge, MA, USA).

#### Ethical considerations

The National Committees for Research Ethics in Norway consider outbreak investigations as public health practice, and approval by the research ethical committee is not needed as long as the objective is to control the outbreak (this is in line with the 1991 International Guidelines for Ethical Review of Epidemiological Studies by the Council for International Organisations of Medical Sciences). Ooutbreak investigations follow the regulations on patient privacy and protection as stated in the Norwegian Law on Communicable Disease Control.

### Microbiological and environmental investigation

#### Patients

Initial isolation of STEC from stool samples was attempted at the primary diagnostic laboratories. Because several of these laboratories routinely only test for *E. coli *O157 using sorbitol MacConkey's (SMAC)-agar, they were early on instructed to examine also for *E. coli *O103 or refer suspect samples or strains to an expert laboratory. All suspected STEC isolates were sent to NIPH for verification, DNA-profiling by MLVA [6] and examination of virulence factors, including the genes *eae, stx*_1_*and stx*_2_, using an octaplex PCR [7]. This PCR method was also implemented to screen multiple colonies from mixed cultures. Isolates which tested positive for *eae*, *stx *or both, were examined further for serotype affiliation and MLVA profile. Since it was observed that the outbreak strain displayed a distinct morphology on Chromocult coliform agar (Chromocult; Merck, UK) [8], this medium was also used to select suspect colonies from mixed cultures.

The Stx2 toxin was subtyped according to the scheme proposed by Persson et al. [9] by full sequencing of the *stxAB*_2 _operon. A few strains were also sent to the WHO Escherichia reference centre at State Serum Institute in Denmark for verification and further characterization.

After an initial Widal agglutination test performed at the NIPH, available sera from cases were sent to the German HUS reference laboratory at the Münster University Clinic to perform IgM/IgG immunoblot assays against serogroup O103 and other important HUS-associated serogroups.

#### Food-products and production sites

The NFSA collected leftover food items from patients' homes and products from shops of purchase. The NFSA also traced the suspected products and their ingredients, sampled raw materials and ingredients and inspected and sampled the producers' premises. Brand labels and batch numbers of items were checked. Samples were tested for the presence of STEC O103 at the Norwegian School of Veterinary Science using modifications of two methods (methods no. 125 and 164) suggested by the Nordic Committee on Food Analysis [10], including automated immunomagnetic separation specific for *E. coli *O103 (Dynabeads STEC/VTEC O103; Invitrogen Corp., Oslo, Norway). Presumptive STEC O103 isolates from the immunomagnetic separation procedure which showed the distinct colony morphology on Chromocult coliform agar [8], were sent to the NIPH for confirmation, virulence factor testing and MLVA typing as specified above.

## Results

### Epidemiologic investigation

#### Case finding

Between February 20 and April 7, 2006, 17 cases were identified: 10 cases of HUS, six cases of diarrhoea and one asymptomatic case (who was the mother of a HUS case). All 10 HUS cases and four diarrhoea cases were hospitalised. One HUS patient died within five days after hospitalisation. Sixteen cases had diarrhoea of which 14 had bloody diarrhoea. Two cases also reported haematuria.

**Figure 1:**
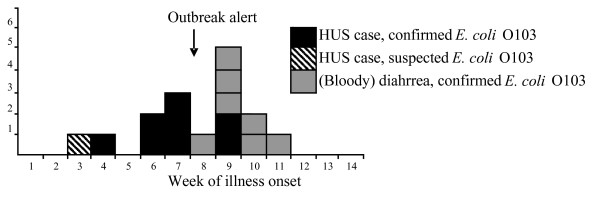
Distribution of cases of haemolytic uraemic syndrome (HUS) and *E. coli *O103 infection by week of onset of symptoms, Norway, January-March 2006 (n = 16).

**Figure 2:**
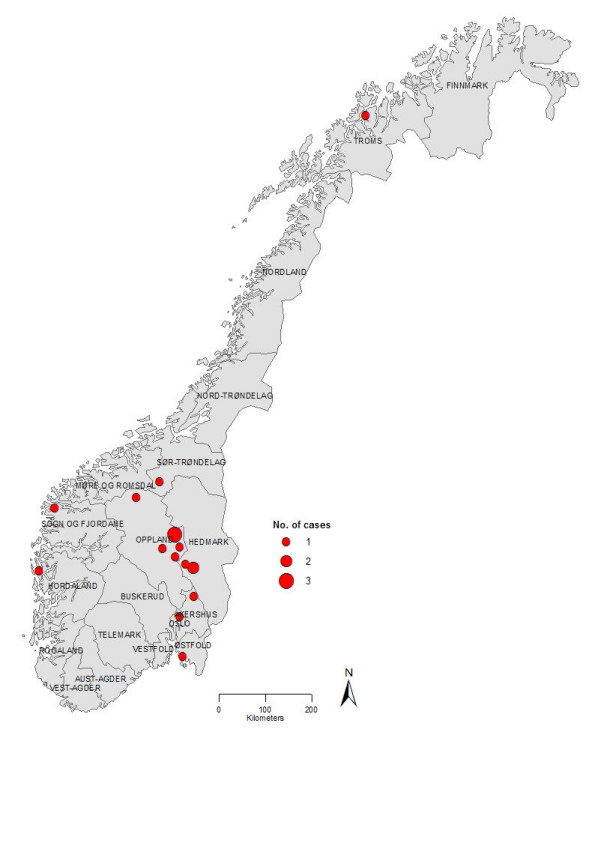
Distribution of outbreak related cases of HUS and *E. coli *O103 infection by county, Norway, January-March 2006 (n = 17).

Dates of symptom onset ranged between January 22 and March 13, 2006, with 15 cases reporting first symptoms between February 12 and March 13 (Figure 1). Ten cases came from mid-Norway, in two neighbouring counties. Among the other cases residing in seven other counties (Figure 2), one had visited one of the first counties the week before illness. Eleven (65%) cases were female, and all cases were children aged 1–11 years, except the asymptomatic case (aged 40) and another adult (aged 18) with diarrhoea only. All HUS cases were less than 9 years old.

#### Hypothesis-generating (pilot) interviews

The interviews of the first six HUS cases were completed on February 22 and revealed no recent travel history, or common exposures to restaurants, events or animal contact. However, some food products were commonly mentioned, including minced meat of one specific brand. By March 14, an additional five cases had been notified. Continued hypothesis-generating interviews with these new cases identified another common exposure; a traditional cured sausage product called 'morrpølse'. This product had been mentioned by only one of the first six patients. However, repeated interviews with the patients' parents specifically asking about possible exposure to this sausage product by visiting friends, relatives or at other places, found that this product could be linked to all cases except one.

#### Case-control study

In the first case-control study, all six HUS cases and six of 18 matched controls had eaten minced beef from producer A (OR = 8.5 p = 0.04). At this stage in the investigation no pathogen was identified in the six HUS cases. The second case control study including all 15 cases known to us at that time and their 45 matched controls identified several meat products to be associated with illness (Table 1). In the final multivariable model, only the consumption of traditional cured sausage ("morrpølse") and eating beefburger at a fast-food restaurant of chain B were independently associated with illness (Table 1).

**Table 1:** Results of case-control study of causes of *E. coli *O103 outbreak in Norway, January-March 2006. The matched analysis includes 15 cases and 45 controls.

Food consumed	No. of cases (%)	No. of controls (%)	mOR	95% confidence interval	p-value	Adjusted mOR	95% confidence interval	p-value
Cured sausage ("morrpølse")	9/14 (64)	6/45 (13)	19.4	(2.4 – 156)	0.005	17.6	(1.6 – 187)	0.018
Beefburger at fastfood chain B*	7/15 (47)	5/45 (11)	14.7	(1.7 – 123)	0.014	12.5	(0.9 – 181)	0.065
Minced beef from producer A	10/14 (71)	20/44 (45)	2.7	(0.8 – 9.5)	0.124			
Lean pork patties	8/14 (57)	9/44 (20)	5.1	(1.3 – 20.4)	0.023			

* Received raw beef patties from producer A

### Microbiological and environmental investigation

#### Patients

Stool or serum specimens were obtained from all 17 cases (Table 2). Nine of the ten HUS cases were confirmed as having an *E. coli *O103 infection, using either stool sample culture (four cases) or serology (five cases). One HUS case had negative results for serogroup O103 by both methods, but was positive with LPS IgM serology for *E. coli *O157. Six cases with diarrhoea and one asymptomatic family contact had a positive stool sample for *E. coli *O103. The serotype of the outbreak strain was identified as *E. coli *O103:H25. All eleven patient isolates were positive for the *eae*-gene (intimin encoding), but Shiga toxin genes could be detected in only two isolates, both with Shiga toxin 2 only. By sequencing, the outbreak strain was allocated to subtype Stx2-O157-EDL933 according to the subtyping scheme suggested by Persson et al. [9]. The patients concerned developed HUS. All outbreak isolates, irrespective of their *stx*-status, belonged to the same MLVA-profile cluster, which were markedly different from the collection of 106 other *E. coli *O103 isolates at the NIPH except for two strains from HUS patients diagnosed in 2003 and 2005 respectively. A dendrogram comparing the isolates from the present outbreak with epidemiologically unrelated O103 strains has been presented previously by Linstedt et al. [6].

**Table 2:** Evidence of *E.coli *O103 infection in HUS cases (10) and diarrhoeal cases (6) and asymptomatic case (1), January-March 2006 (n = 17)

	Diarrhoea/bloody diarrhoea	HUS	Stool culture for *E.coli *O103	*eae*-gene	*stx*_1_/*stx*_2_	MLVA outbreak profile *	Antibody titre against outbreak strain**	IgG/IgM Immunoblot *E.coli *O103
1	+/+	+	+	+	-/-	+	320	+/+
2	+/+	+	+	+	-/+	+	-	-/+
3	+/+	+	-	NA	NA	NA	160	+/+
4	+/+	+	-	NA	NA	NA	320	+/+
5	+/+	+	-	NA	NA	NA	320	+/+
6	+/+	+	-	NA	NA	NA	640	NT
7	+/+	+	-	NA	NA	NA	640	+/+
8	+/-	+	-	NA	NA	NA	-	-/-***
9	+/+	+	+	+	-/+	+	NA	+/+
10	+/+	+	+	+	-/-	+	NA	NT
11	+/+	-	+	+	-/-	+	NA	NT
12	+/+	-	+	+	-/-	+	NA	NT
13	+/+	-	+	+	-/-	+	NA	NT
14	+/+	-	+	+	-/-	+	NA	NT
15	+/+	-	+	+	-/-	+	NA	NT
16	+/-	-	+	+	-/-	+	NA	NT
17	-/-	-	+	+	-/-	+	NA	NT

* MLVA profile 07-03-00-05-00-07-01 [6].

** The Widal test was performed against the isolate of case no. 2 defined as the outbreak strain

*** Also tested against serotype O157.

Test results: IgG-/IgM + NT: not tested, NA: not available.

#### Food-products and production sites

Two days after the product recall, *E. coli *O103 with the outbreak MLVA-profile was isolated in unopened packages of the recalled cured "morrpølse". This product was produced only in facility A which had distributed 3.5 tonnes nationwide between December 2005 and March 20, 2006. The sausage contains mutton and pork in addition to starter culture, spices and preservatives. The curing process takes approximately three weeks. Low pH, low water activity and an added bacterial culture are the main barriers against pathogen growth. No heat treatment is included in the production process. Among the different ingredients of the incriminated sausage, *E. coli *with the distinct characteristics of the outbreak strain was only recovered from mutton.

From facility A, the outbreak strain was also traced to production plant B (frozen mutton), then to slaughterhouse C (mutton) and then to four farms (sheep).

### Public health action

On February 24, based on our first report, the NFSA issued a public warning against minced beef from producer A and the producer withdrew all minced beef from the production facility they believed to be the origin of the suspected food based on the geographical distribution of the cases. The importance of good kitchen hygiene and proper cooking of minced meat was stressed by health authorities.

On March 20, based on the extended case-control study, the NFSA withdrew the incriminated cured sausages ("morrpølse") from the market and warned the public against eating these products produced between December 1, 2005 and March 20, 2006. The production facility A was closed, and then cleaned and inspected before production could resume. Sheep slaughtering techniques were changed to reduce contamination.

## Discussion

We have reported an outbreak of haemolytic uremic syndrome caused by a highly virulent strain of Shiga toxin 2 producing *E. coli *O103:H25. Dry cured sausages were identified as the vehicle of infection, as evidenced by results of a case control study and subsequent isolation of the outbreak strain in the product.

We also found a strong association between illness and consumption of beefburger at a fast food restaurant chain. The burgers were produced in the same facility as the initially suspected minced beef. The first case-control study that implicated minced beef from producer A included only six cases. Due to the emergency of the outbreak, these cases were used for both generating and testing the hypotheses, which might have introduced an information bias. Later in the investigation, after the suspicion about minced beef was announced, there were many reports about the "burger-bug" in the media. We believe that recall bias may have caused the association with beefburgers from chain B in the second larger case-control study.

Long after the product had been withdrawn, it was discovered through immunoblot analysis that one case (no 8, table 2) probably was infected by another *E. coli *than the outbreak strain, and thus was proably not related to the outbreak. We have still presented the analysis including this case (table 1) because it fulfilled the case definition used during ther investigation, and it was this analysis that formed the basis for decision making at the time. This illustrates the dillemma of choosing between a sensitive and a specific case definition during an outbreak investigation.

This investigation illustrates that even when it is considered necessary to warn customers about suspected products early in the investigation, it is important to continue investigating alternative sources vigorously until the source can be confirmed and no more cases occurs after the source is removed.

Genotyping of the isolates was pursued using a recently published MLVA assay, which is designed for differentiation within all *E. coli *serotypes [6]. MLVA is generally considered to be a robust and fast typing method with high discriminatory power [6,11]. Prospective MLVA subtyping of STEC may be used to detect clusters of related cases and ascertain the source of infection. The high discriminatory power of the MLVA method for O103 as well as for *E. coli *in general, has been documented by Lindstedt et al. [6].

The outbreak was detected by clinicians reporting a cluster of HUS cases, and not by an increase in laboratory verified *E. coli *O103 infections. The same has been the case in a few other reported STEC outbreaks; one STEC O111 outbreak in Australia [12], some German STEC O157 (sorbitol-fermenting) outbreaks [13,14] and two Italian HUS outbreaks involving non-O157 strains [15,16]. Most of these outbreaks would have been missed by screening faecal samples using sorbitol MacConkey's (SMAC) agar as this medium is intended for the detection of of sorbitol-negative O157 strains. Underreporting of non-O157 *E. coli *strains is assumed due to the current laboratory methods used in many countries [2,16], including Norway. Therefore, surveillance of diarrhoea-associated HUS may be needed for early detection of STEC outbreaks [17].

STEC O103:H2 is one of the most frequently isolated non-O157 serotypes in many European countries and can cause severe illnesses comparable to those caused by serogroup O157 [18]. It was first identified as a causative agent of HUS in 1992 [19], however cases are mainly sporadic and outbreaks of STEC O103:H2 have rarely been reported. One family outbreak was reported in Japan [20] and one day-care centre outbreak in Argentina [21], both caused by *stx*_1_-positive STEC O103. The sources were not identified. The serotype of the present outbreak – O103:H25 – is extremely rare. A few sporadic cases have been reported [22,23], and where the toxin profile has been reported, all have been *stx*_1_-positive. Outbreaks or sporadic cases caused by *stx*_2 _positive *E. coli *O103:H25 have to our knowledge never previously been identified.

More than half the patients developed HUS. In spite of widespread testing of contacts and enhanced laboratory procedures during the outbreak, very few additional mild cases were identified. Only one family member of a HUS case reported symptoms. An increase in non-outbreak related STEC infections in patients with milder symptoms were reported, which could indicate that the sensitivity of testing improved during and after the outbreak. Thus, we believe that the outbreak strain was particularly virulent. Also in some other outbreaks, a similar high proportion of HUS cases have been observed [13,14,24]. STEC strains, independent of serogroup, that contain the *eae *gene and produce Stx2 are much more likely to cause severe disease and HUS than are those that produce Stx1 [25-27]. In addition, sequencing of the *stx*_2 _operon showed that it encoded a Stx2 variant that is more often associated with HUS than other subtypes [9]. The reason why *stx*-genes could be demonstrated in only two of the outbreak isolates, despite the fact that the isolates were closely related genetically, will be the subject further research. Bielaszewska et al studied patients with HUS hospitalized in Austria and Germany, and found that at the time of microbiological analysis, 5% of HUS patients shed no longer the causative STEC, but excreted *stx*-negative derivatives that had lost *stx *during infection [28]. Other studies have also shown that STEC may loose the *stx*-genes during infection or subcultivation [29-32].

A Danish study found that serogroups O157 and O103 were independent risk factors for bloody diarrhoea [33]. Earlier reports have suggested that *E. coli *O103:H2, can be regarded as an emerging foodborne pathogen [34] and warned that additional uptake of *stx*_2_-phages by *E. coli *O103 can result in the emergence of a strain with increased virulence as has occurred with *E. coli *O26 [18,34].

The two cases diagnosed with *E. coli *O103:H25 with the same MLVA- and virulence profile in 2003 and 2005 may indicate that this strain has been present over a longer time period in Norway. However, it is not known if these cases were related to cured sausage or other products containing mutton. Earlier reports have shown that Norwegian sheep may carry human-pathogenic STEC [35], including STEC O103 [36]. Mutton products were recently associated with an outbreak of STEC O26 in France [37].

Fecal contamination of carcasses is practically unavoidable, and STEC are able to survive the fermentation, drying, and storage stages of sausage production [38], as shown in experimental studies [39-41]. Several STEC outbreaks caused by dry fermented sausages have been described [12,42-45]. These products are often made of combinations of meat mixed with spices and curing materials that then undergo fermentation and drying. In general, it is believed that short ripening time may lead to incomplete fermentation and thus to conditions (e.g. pH) that still allows growth or survival of enteric pathogens. Since these sausages are eaten raw and the infectious dose for STEC is very low, the producer must assure microbiologically safety. The products are believed to constitute a 'medium' risk hazard for STEC infection [39,46,47], and the US Food Safety and Inspection Service has developed guidelines [48] for sausage manufacturers to validate processes to ensure a 5 log10 unit (5D) reduction in counts of STEC O157:H7, for instance by heating the final product. Still, we believe that the consumers should be made aware of the inherent risk associated with these products [46].

We do not know why STEC survived the curing process in the incriminated "morrpølse". An evaluation committee suggested that there should be stricter requirement on hygienic quality for raw meat used for such products. In addition, the producer should document the effectiveness of the production process in reducing the level of pathogens if present in raw meat or other ingredients.

## Conclusion

In summary, we report an outbreak of infections with a rare strain of STEC (O103:H25, *stx*_2_-positive). More than half of the diagnosed patients developed HUS, indicating that the causative organism is particularly virulent. The source of the outbreak was cured sausages produced in one processing plant. The outbreak created a huge media attention, raised concerns about food safety and had large economical consequences for the producer. The potential for future outbreaks warrants a re-evaluation of the microbiologic health-hazards in the production of cured meat sausages.

## Competing interests

The author(s) declare that they have no competing interests.

## Authors' contributions

PA was responsible for leading the outbreak investigation. BS, KN and HME were principal investigators of the epidemiological investigation and BS drafted the manuscript. JL and GK were responsible for the microbiological investigation of the outbreak. BAL and LTB carried out the genotyping and virulence factor testing of patient and environmental isolates. All authors have read and approved the final manuscript.

## Pre-publication history

The pre-publication history for this paper can be accessed here:


